# Awakening Canadians to ageism: a study protocol

**DOI:** 10.1186/s12912-021-00713-0

**Published:** 2021-10-09

**Authors:** Sherry Dahlke, Kathleen F. Hunter, Mary T. Fox, Sandra Davidson, Nicole Perry, Laura Tamblyn Watts, Lori Schindel Martin, Jeffrey I. Butler, Christy Raymond, Alison L. Chasteen, Lynn McCleary, Veronique Boscart, Elaine Moody

**Affiliations:** 1 Faculty of Nursing, University of Alberta, Edmonton Clinic Health Academy, 11405 - 87 Ave NW, Edmonton, AB T6G 1C9 Canada; 2 School of Nursing, Faculty of Health, York University Centre for Aging Research and Education, HNES suite 343, 4700 Keele St, Toronto, ON M3J 1P3 Canada; 3 Faculty of Nursing, University of Calgary, 2800 University Way N.W., 2500 University Drive NW, Calgary, AB T2N 1N4 Canada; 4 National Programs, HelpAge Canada, 1300 Carling Avenue, Ottawa, ON K1Z 7L2 Canada; 5 CanAge, Factor – Inwentash Faculty of Social Work, University of Toronto 246 Bloor St West, Toronto ON M5S 1V4, Toronto, ON M5S 1V4 Canada; 6 Canadian Gerontological Nursing Association, PO Box 64009, Royal Bank Plaza, Toronto, ON M5J 2T6 Canada; 7 School of Nursing, York University Centre for Aging Research and Education, Faculty of Health, York University; Faculty of Nursing, University of Alberta, HNES suite B05, 4700 Keele Street, Toronto, ON M3J 1P3 Canada; 8 Faculty of Nursing, MacEwan University, 5-111C, Robbins Health Learning Centre, 10910-104 Ave NW, Edmonton, AB T5J 4S2 Canada; 9 Department of Psychology, University of Toronto, 100 St. George Street, Toronto, ON M5S 3G3 Canada; 10 Faculty of Applied Health Sciences, Brock University, 1812 Sir Isaac Brock Way, St. Catharines, ON L2S 3A1 Canada; 11 School of Health and Life Sciences, Conestoga College Institute of Technology and Advanced Learning, 299 Doon Valley Drive, Kitchener, ON N2G 4M4 Canada; 12 School of Nursing, Dalhousie University, Room N19 Forrest Bldg., Dalhousie University, University Avenue, PO Box 15000 5869, Halifax, NS B3H 4R Canada

**Keywords:** Ageism, Older Canadians, Knowledge mobilization, E-learning activities

## Abstract

**Background:**

Making fun of growing older is considered socially acceptable, yet ageist humour reinforces negative stereotypes that growing old is linked with physical and mental deterioration, dependence, and less social value. Such stereotypes and discrimination affect the wellbeing of older people, the largest demographic of Canadians. While ageism extends throughout professions and social institutions, we expect nurses—the largest and most trusted group of healthcare professionals—to provide non-ageist care to older people. Unfortunately, nurses working with older people often embrace ageist beliefs and nursing education programs do not address sufficient anti-ageism content despite gerontological nursing standards and competencies.

**Methods:**

To raise awareness of ageism in Canada, this quasi-experimental study will be supported by partnerships between older Canadians, advocacy organizations, and academic gerontological experts which will serve as an advisory group. The study, guided by social learning theory, will unfold in two parts. In Phase 1, we will use student nurses as a test case to determine if negative stereotypes and ageist perceptions can be addressed through three innovative e-learning activities. The activities employ gamification, videos, and simulations to: (1) provide accurate general information about older people, (2) model management of responsive behaviours in older people with cognitive impairment, and (3) dispel negative stereotypes about older people as dependent and incontinent. In Phase 2, the test case findings will be shared with the advisory group to develop a range of knowledge mobilization strategies to dispel ageism among healthcare professionals and the public. We will implement key short term strategies.

**Discussion:**

Findings will generate knowledge on the effectiveness of the e-learning activities in improving student nurses’ perceptions about older people. The e-learning learning activities will help student nurses acquire much-needed gerontological knowledge and skills. The strength of this project is in its plan to engage a wide array of stakeholders who will mobilize the phase I findings and advocate for positive perspectives and accurate knowledge about aging—older Canadians, partner organizations (Canadian Gerontological Nurses Association, CanAge, AgeWell), and gerontological experts.

Contributions to the literature

- Ageism in Canada extends through many organizations and institutions, including healthcare. This study will build knowledge on the effectiveness of e-learning approaches in dispelling ageist misconceptions amongst student nurses.
- This study will generate evidence on the effectiveness of the three e-learning activities on student nurses’ perceptions of older people.
- This study’s partnership approach to knowledge mobilization can guide researchers seeking to involve key stakeholders and community-members in the development and implementation of strategies to improve healthcare education and delivery, enacting positive social change.

## Background

### Understanding ageism

Canada has more people aged 65 and older than it has children 14 and younger [1], with ageism impacting many of these older Canadians. Butler [2] defines ageism as negative perceptions attributed to a population based on its age. Ageism includes negative attitudes towards older people, aging, and the aging process, as well as discrimination that is either individual or systemic [3]. Stereotypes of growing old include increasing illness, isolation, poverty, and depression contribute to ageism [4]. Further, negativity towards older people is accepted, without social sanctions [5], with the result that it flourishes in Canada. Ageism often emerges “insidiously, veiled in claims of ‘best interest,’ or humor” ([6] p. 644), as a socially acceptable form of discrimination. Most older Canadians live independently in the community and growing numbers continue to work after the age of 65 [7, 8]. Unfortunately, they experience ageism when purchasing goods and services [9], in their familial relationships [10] and in healthcare institutions [11].

#### Impacts of ageism

Research has demonstrated a negative association between age discrimination and older people’s well-being [12] and self-esteem [13], and a positive association with depressive symptoms [14]. This suggests that negative stereotypes about aging can become internalized [13, 14], so that conscious or unconscious ageist perceptions and stereotypes influence the cognitive and/or physical functioning of older people and erode their will to live [15]. Various international studies have reported the deleterious effects of ageism: healthcare costs in the United States due to health-related illness from ageism are estimated at $63 billion a year [16]. European-based studies have reported that older people are treated negatively and undervalued [17, 18]. A 2008 Canadian study involving people of all ages reported that, regardless of culture and religious philosophy, Canadians view older people as “less vital” and “non-accommodating” ([19] p. 28); the study also revealed that younger Canadians respect older people but want to avoid them.

#### Aging in a nursing context

Palmore [20] has called ageism a “virus” ingrained into cultures, so pervasive that even older people have negative stereotypes of aging. Thus, it is not surprising that ageism, in the form of poor care of older people, persists within healthcare, despite initiatives to improve their care and status [6]. Healthcare systems are designed for younger people, emphasizing efficiency and quick turnover, and do not consider the complexity of older people’s health and social concerns [11, 21]. This ageist perspective extends to the nursing profession, the largest group of healthcare professionals [22], with many nurses believing that working with older people is of low occupational status [23]. Scholars have suggested that nurses’ ageist stereotypes exist because they see older people at their most vulnerable and dependent [24–26]. Additionally, when nurses hold negative stereotypes of cognitive and physical decline of older people [3]—and believe that working with older people is simple [24–26]—they fail to adequately assess and manage older people’s healthcare concerns, leading to negative consequences [25, 27–31]. Conversely, when nurses have positive perceptions of older people, they are more likely to recognize older people’s health and social needs and meet them appropriately [19, 32].

A recent review of gerontological competency frameworks in Canadian health and social service education suggests the need for greater gerontological competencies across professions [33]. Canadian gerontological experts have developed gerontological competencies for nursing programs [34], and professional gerontological nursing standards and competencies to meet best practice expectations [35]. Unfortunately, these competencies have not been consistently incorporated into accreditation processes or curriculum revision initiatives and Canadian health and social service faculty are often unaware of published gerontological competencies that could aid them in curricular evaluation and optimization [36]. Barriers to integrating gerontological competencies include insufficient faculty expertise in gerontology and little recognition that gerontological competencies are needed [36]. If ageism was acknowledged, there would be a stronger impetus for implementing these competencies, thereby better serving older Canadians.

Against this background, scholars have identified that nurses are graduating with inadequate knowledge about working with older people [37–40], including how to care for them [26, 41]. Students observe what practitioners say and how they model care provision to older people, which can have a lasting impact on professional learning and socialization [42] and shape how student nurses learn to work with older people. Unfortunately, practicing nurses who demonstrate ageist behaviours towards older people unwittingly contribute to the professional socialization of students [24]. Our prior work revealed that practicing nurses and nurse educators often perpetuate student’ negative perceptions and pass on negative practices to the next generation of nurses [43]. For instance, when students witnessed nursing staff not listening to older people or neglecting to attend to their needs, the students inferred that these patients were not a priority, and that older patients’ concerns need not be addressed. Overall, students were not prepared for the complexity of the aging population, due to minimal curricular focus on older people, and lack of strong gerontologic role models [43]. Still, students wanted to learn more about aging and expressed interest in web-based and/or simulated learning activities. In another study, newly graduated nurses likewise identified the need for web-based learning activities that enhance knowledge about working with older people [44].

A critical review of the literature examining nursing education related to older people suggests that nursing programs have focused on integrating content about older populations into curricula, but not on the social aspects of learning processes that expose students to ageist perspectives and practices [45]. Certainly, students come from a wide range of cultures that contribute to socialization and beliefs about growing old, but they are also exposed to the negative stereotypes about aging embedded in nursing and healthcare cultures. Nursing programs reinforce uptake of these negative stereotypes and perpetuate ageism by privileging technical knowledge over gerontology-focused care [46]. Few nurse educators have gerontological expertise, which would enable them to actively dispel negative perceptions within nursing programs [37–39, 47]. Taken together, the dearth of gerontology-prepared nursing faculty, insufficient gerontological content, and learning experiences in nursing programs that perpetuate stereotypes of aging create negative care experiences for older Canadians.

### Study objectives and significance

A partnership with Canadian organizations that advocate for older people and the involvement of gerontological experts is needed to bring this awareness to the public and professionals that work with older people. Accordingly, we have the following three objectives:

1. To test if we can displace ageist perspectives, we will use student nurses as a test sample to determine if innovative e-learning activities aimed at dispelling aging myths improving students’ perceptions of older people and reduces negative stereotypes of growing old.
2. To develop short-, medium-, and long-term strategies, based on the test results, aimed at dispelling negative stereotypes about aging in professional groups that serve older people and the Canadian public. An advisory group consisting of older Canadians and representatives from CanAge, HelpAge, Canadian Gerontological Nursing Association (CGNA), and academic gerontological experts will be assembled to tackle this challenge and identify appropriate methods to bring positive messages about aging to Canadians.
3. To implement the short-term strategies developed by the advisory group. The specifics of the implementation plan will solidify as findings from testing the e-learning activities with student nurses and discussion with the advisory group are developed into strategies.

This project will improve how older people are treated in healthcare by creating awareness of ageism towards older Canadians, defined as people aged 65 and older [48]. Palmore [20] purports that the best way to reduce ageism is to bring awareness to it—our overarching aim—by developing strategies to dispel negative stereotypes about aging. We choose to address negative aging stereotypes in student nurses based on the findings of our previous research, but also because nurses, who are influential in healthcare teams, can model positive practice. If student nurses’ perceptions towards older people can be improved, other professionals and the Canadian public’s perceptions may be similarly improved to enhance service to older Canadians. Importantly, implementing the short-term strategies we develop will rapidly mobilize the initial research findings, but the project also has the potential to leverage relationships amongst the partners to implement medium- and long-term strategies.

### Theoretical framework: social learning theory

Bandura’s social learning theory which proposes that people learn new behaviours through observations in their social environments [32] will guide each step of this study. Social learning theory illuminates how people learn in society and proposes that behaviour and beliefs are often attributed to underlying forces that can be modified or eliminated with a new stimulus or learning approach. Student nurses, for example, learn in complex social environments that straddle classrooms and clinical settings, where they are exposed to healthcare professionals who may unconsciously be perpetuating negative behaviours towards older people [49]. Social learning theory will spur the project team to be attentive to the multiple ways that learning occurs (e.g., reading information, hearing information verbalized, observing the behaviour of others, etc.) and the importance social context plays in learning. The theory will direct our development of knowledge mobilization strategies to dispel negative stereotypes amongst Canadians and test student nurses’ perceptions about older people, following the e-learning activities, by focusing on the social aspects of how people learn, i.e., through the images they see, the messages they hear, and the behaviours related to ageing and older Canadians that are modeled for them. We propose that as student nurses’ perceptions improve and they acquire accurate knowledge, the current inadequate assessment and management of older people’s healthcare concerns, embedded as they are with ageist stereotypes, will dissipate [27, 29, 31]. Social learning theory will additionally guide us in analyzing the data, interpreting the findings, and knowledge mobilization, through careful attention to social contexts that influence how people perceive and behave towards older people.

## Methods/design

To raise awareness of ageism in Canada, this quasi-experimental study, supported by partnerships with older Canadians, advocacy organizations—Canadian Gerontological Nurses Association (CGNA) —gerontological nurses, and academic gerontological experts, will examine the effectiveness of e-learning activities on ageist attitudes then develop and implement strategies to combat such attitudes.

### Innovative learning approaches

In Phase one of this project, we will use innovative methods such as e-learning—education delivered on a digital device [50]—that incorporates gamification, videos, and simulation to convey positive perceptions of older people and non-ageist behaviours by healthcare professionals. A systematic review of the literature [51] found that that e-learning is an effective strategy for improving professionals’ knowledge, competence, and behaviours. Further, our literature review of web-based learning for nursing education revealed that gamification, simulation, and web-based virtual worlds are avenues that improve knowledge transfer and satisfaction with e-learning [52]. Gamification is defined as “the use of game design elements in non-game contexts” [53] p. 9 and is an engaging, interactive, and stimulating way to learn [54]. Nursing students identified that gamification increased their satisfaction with learning, stimulated creative thinking, and improved decision-making [55]. Virtual reality and self-directed e-learning, followed by practice in a virtual world, are also effective ways of acquiring new knowledge and skills [56–59].

Using e-learning activities to facilitate students’ development of positive perceptions of aging and gerontological expertise does not require instructors with academic expertise in those domains. Such change opens the possibility of improved interactions and outcomes for older Canadians. Moreover, if e-learning is an effective method for student nurses, it may be effective with other healthcare professions as well.

### Phase I (months 1 to 8): dispelling stereotypes (objective 1)

A quasi-experimental pre-post-test design will be used to test if the e-learning activities improve student nurses’ perceptions about older people—in particular, by dispelling stereotypes and providing positive role models for working with older people.

#### Setting and sample

The study will be conducted at two Canadian nursing programs to provide geographic variability of eastern and western Canada (York University and the University of Calgary). We estimate 1300 eligible students at York and 825 at U of C (total of 2125). Assuming a conservative 30% response rate, we anticipate a sample size of ~ 640 students. The sample size is estimated to attain adequate power to detect a small effect size of the e-learning intervention on student nurses’ perceptions about older people. Using Cohen’s [60] criteria of an anticipated small effect, a comparison among pre-and post-test scores and an α of .05 β set at .80, a minimum sample size of 393 is needed.

To maximize the response rate, Dillman’s [61] total survey method will be used. This method involves emailing eligible students a link to the e-learning activities and the survey. Non-responders will receive up to four email reminders, at two-week intervals after the first e-mail (i.e., three, five, seven, and nine weeks). A $10.00 gift card will be offered as an incentive for participating in each learning activity; this has been identified to increase survey completion rates [62].

#### Learning activities

To dispel negative stereotypes and ageism among student nurses, we will use three e-learning activities developed in our prior research [43] and previously tested with student nurses [63]. The topics of the activities were selected based on our prior work and target student nurses’ most urgent gerontological learning needs [43]. We will use games, videos, and simulations that model positive behaviours in the e-learning activities to spark interest and capture participants’ attention.

*Learning activity 1* will challenge negative stereotypes by providing information about healthy older Canadians and how to communicate with older people. Our previous research identified that student nurses lack knowledge about older people and how to communicate with them, which contributes to ageist perspectives [43]. We estimate participants will take 90 min to complete this activity.

*Learning activity 2* will focus on cognitive impairment (delirium, dementia, and depression), including positive modelling on how to manage anxiety and aggression in older people with these conditions. Previous research identified that student and practicing nurses do not feel equipped to work with older people with dementia [28, 43, 64, 65]. We estimate participants will take 3 h to complete this activity.

*Learning activity 3* will focus on dispelling stereotypes about older people as dependent and incontinent. Continence is a complex health issue influenced by cognition and physical ability, including mobility [66, 67]. Students nurses often hold negative stereotypes that incontinence is inevitable and believe it is not part of their job to help older people manage continence [68–70]. We estimate participants will take 90 min to complete this activity.

#### Data collection

Student nurses will complete a pre- and post-test using Burbank’s [71] measure of their perceptions about working with older people and the Ambivalent Ageism Scale (AAS) [4]; participants will also complete a post-test survey on their opinions of the learning activities. The pre- and post-e-learning activities will include the following three self-response scales:

Burbank’s Perceptions About Older People quiz [71] is a nine-item, 5-point Likert-type scale. The total score is derived by taking the mean of the responses. The higher the score out of five, the more positive the perception. This measure has demonstrated strong reliability and validity in previous studies [64, 72].

The Ambivalent Ageism Scale (AAS) is a 13-item, 7-point Likert-type scale, which measures benevolent and hostile attitudes towards older people [4]. The AAS provides a means to examine the complexity of ageism, including both positive and negative stereotypes measured by the benevolent (9 items) and hostile (4 items) ageism subscales, respectively. The subscale scores are derived by taking the mean of the responses. The overall scale has high test-retest reliability (r = .76) and internal consistency (a = .91). The reliability for the two subscales also demonstrated high test-retest reliability with benevolent (r = .71), hostile (r = .80) and internal consistency for the benevolent and hostile subscales (both α = .89).

*Post-learning activity survey* will be completed after each learning activity. The survey will include demographic questions and 5-point, Likert-type scale items measuring their perceptions about the learning activity.

To control for possible order effects in the survey responses, the sequence of the e-learning activities will be randomized using a computer-generated randomization scheme.

#### Data analysis

Descriptive statistics will be used to describe participants’ average standing on all measured variables. We will use paired t-tests (after testing for assumptions) to compare participants’ pre- and post-test scores on Burbank’s quiz and the AAS subscales.

### Phase II (months 8 to 36): developing anti-ageism strategies (objective 2)

Our advisory group will include older Canadians, representatives from CanAge, HelpAge, and the CGNA, as well as gerontological experts. With help from our partners CGNA, AgeWell and CanAge, we will recruit older Canadians who represent rural and urban populations from different provinces. Through Appreciative Inquiry, which emphasizes positive change, the advisory group will examine the findings from the test case of student nurses’ e-learning activity [73]. The findings from the test case of student nurses will promote discussion of the possibilities of using similar strategies with other healthcare professional groups or other populations.

In bi-monthly virtual meetings over the 36 months of the project, group members will discuss where ageism is prevalent in the world around us. Through the meetings and examination of the test data, group members will reflect on ageism in Canada to develop short-, medium-, and long-term strategies aimed at dispelling ageism among professional groups that work with older Canadians, as well as the Canadian public. Anticipated outcomes include open conversations about ageism in our society and how it affects services delivered by Canadian health and social agencies. Four steps characterize the Appreciative Inquiry approach [73] we will employ:

1. “Discovering the core issues” will occur in the first 8 months and will include examining the findings from the test case of the e-learning activities with student nurses, as well as conducting group conversations about what participants in the advisory group view as salient issues.
2. “Dreaming about what might be” will occur from the 9th to 16th month, and will include gathering the advisory group’s vision for an ideal future—when ageism is recognized, and stereotypes are dispelled.
3. “Designing strategies that facilitate a preferred future” will occur from the 17th to 26th month. The advisory group will develop short-, medium- and long-term strategies to facilitate positive perspectives and approaches to ageing and older people among professional groups and the Canadian public (using the student test with e-learning activities as a starting point for what might work with other groups).
4. “A plan to implement the strategies” will be incorporated in the final phase of the project that will involve the advisory group developing and implementing the short-term strategies. The fourth step of Appreciative Inquiry aligns with our final objective, and thus constitutes final part of the research project.

### Our partnership approach

A partnership approach is integral for this project because it brings together a diverse group of older Canadians, leaders from CGNA, AgeWell and CanAge, organizations that advocate for older Canadians, and academic gerontological experts to engage in an inter-disciplinary, cross-sector initiative. The makeup of this partnership will ensure that the strategies developed are situated within the norms and practices of end-users. Our approach also provides opportunities to engage older Canadians and develop partnerships that may facilitate future research. It is only with this diversity that we can develop effective short-, medium-, and long-term strategies aimed at dispelling ageism in Canada. This partnership is needed to evaluate whether the data from the test of e-learning activities with student nurses (a) is appropriate for use in dispelling negative stereotypes about aging with other professionals and the Canadian public, and (b) can be used to generate other possibilities for developing strategies aimed at dispelling ageism.

Importantly, the partnership provides the opportunity for researchers to learn from older Canadians as well as the leaders of organizations that advocate for and support older Canadians. Moreover, the partnership mobilizes a wealth of research knowledge from the academic gerontological experts. The partners’ and academic gerontological experts’ existing networks and contacts will facilitate knowledge mobilization, and we anticipate that future research flowing from this project will be enriched by the advisory group’s partnerships and discussions.

### Knowledge mobilization

Our advisory group will develop short-, medium-, and long-term strategies designed to dispel ageism. The short-term strategies (described below) will be implemented as part of this project. Working collectively will foster relationships among the partner organizations and academic gerontological experts to facilitate the mobilization of the short-term strategies, as well as enabling future partnerships to mobilize the medium- and long-term strategies (which we will do in future projects). Knowledge mobilization will occur in the third year of the project, as the first two years will be occupied with identifying the issues, examining the data from the test of the student nurses’ e-learning activities, and developing the strategies themselves.

We will be guided by the advisory group to identify the most relevant short-term strategies and ensure that messages used to dispel ageism and the findings from the e-learning activities are widely shared with professional groups that work with older people, as well as Canadians more broadly. Avenues we expect to pursue include development of a website to host publications, infographics, podcasts, publications, and game-based e- learning activities emerging from this study, which will be accessible to all Canadians; publications in *The Conversation Canada,* a website that publishes academic stories for a lay audience; development of podcasts showcasing stories about older people and describing their experiences with age discrimination; targeting relevant media, such as radio and tv, with accounts that highlight ageism in Canada; and, using Twitter and Facebook to send messages aimed at dispelling ageism. We will create an animated narrative vignette (which involves using text and pictures to record conversation in real time) of one of our final advisory meetings to disseminate messages to audiences such as older people, the general public, or school-age children. We will also use game-based approaches, which are effective in creating opportunities for anti-ageism messages that focus on specific target populations [74, 75] to target professional groups and school-aged children to dispel myths about aging, and public talks by partners and academic gerontological experts summarizing our findings and the importance of dispelling ageism at venues such as community centres and at organizations like the Society for Aging in Greater Edmonton (SAGE) and the Canadian Association of Retired People (CARP). We will also link the project website to those of CanAge, HelpAge and CGNA to facilitate distribution of the study findings and implementation strategies to their members through their website, certification programs, and presentations at their bi-annual conferences. Notably, academics, nurse educators, and stakeholders in health and social agencies all attend CGNA conferences. The Canadian Gerontology Association (CAG) includes health and social professionals and holds an annual conference to examine research related to ageing, which gives us the opportunity to disseminate information about our e-learning results and anti-ageism messages. Additionally, our academic gerontological experts will collaborate to develop policy briefs to inform the Minster of Education and Canadian Association of Schools of Nursing of our findings and strategies needed to dispel ageism in schools across Canada.

## Discussion

Ageist stereotypes and discrimination are pervasive in Canadian Society and undermine the health and well-being of older people. To address this issue, this project will raise awareness of ageism in Canada and challenge ageist stereotypes and practices in nursing, other healthcare professions, and the public. In this protocol, we have summarized our objectives, theoretical and methodological approach, and knowledge mobilization plan. The findings will have direct relevance to nursing programs across Canada, continuing education initiatives for practicing nurses, as well as anti-ageism education for other groups of healthcare professionals and the public.

Student nurses will learn to challenge, rather than perpetuate, negative myths about ageing and will acquire urgently needed gerontological competence. Student nurses will learn to avoid unconsciously re-articulating ageist beliefs or behaviours in their nursing care. They will develop more positive perceptions of older people, as well as accurate knowledge of aging and common challenges faced by this population. The Canadian Association of Schools of Nursing and the Canadian Ministry of Education will be engaged in knowledge mobilization strategies with their members based on the findings. Moreover, our academic gerontological experts represent universities across Canada and are well situated within those organizations to disseminate the findings. We will work closely with nursing faculties to develop their expertise and understanding of gerontological competencies through the e-learning activities. The result will be improved experiences in healthcare settings and better health outcomes for older Canadians.

Project partners—AgeWell, CanAge, and CGNA—will all benefit from participating in an interdisciplinary, cross-sector project. Their participation in the advisory group will enhance their understanding of the prevalence of ageism in student nurses as well as effective strategies to dispel negative stereotypes of aging in other professionals that work with older people and the Canadian public. This understanding will improve the care that health professionals provide to older people. The CGNA will have access to cutting-edge e-learning activities to better equip their membership with accurate information about ageist practices of older people they must be aware of and push against when service as clinical preceptors for undergraduate nursing students within their practice settings. This will help the CGNA to dispel ageist views and promote anti-ageist competencies and advocacy, as aligned with their national standards, which will ultimately positively affect older Canadians.

The academic gerontological experts will also benefit from collaborating with one another, the partners, and older Canadians, and have opportunities to develop new research partnerships. The experts will learn from one another’s unique programs of research and research experience, and new partnerships among leading scholars from across Canada will be forged.

Finally, at the broadest level, our partnerships will facilitate ongoing work in dispelling Canadians’ negative stereotypes of aging. Since older people represent the majority of the public, they will benefit from improved understandings about aging across the population as a whole, as well as from improved outcomes due to better service and care. Strategies to dispel negative stereotypes in the Canadian public will be developed, along with an implementation plan to guide their application. Public discourse catalyzed by our knowledge mobilization strategies, such as podcasts about how negative perceptions about aging affect older people, will open doors to frank conversations about ageist perceptions and practices within Canadian society. The messages that the advisory group develops to dispel ageism will further support honest and transparent public conversation, which we anticipate will move Canadians forward in eliminating ageist practices.

## Data Availability

The e-learning activities: (1) Understanding and Communication with older people; (2) cognitive challenges and (3) continence and mobility are available upon request. The corresponding author, Prof. Sherry Dahlke, should be contacted regarding access to any data from this study.

## Abbreviations

AAS: Ambivalent Ageism Scale
CARP: Canadian Association of Retired People
CAG: The Canadian Gerontology Association
CGNA: Canadian Gerontological Nursing Association
SAGE: Society for Aging in Greater Edmonton
